# Interleukin 27 polymorphisms in HCV RNA positive patients: is there an impact on response to interferon therapy?

**DOI:** 10.1186/1471-2334-14-S5-S5

**Published:** 2014-09-05

**Authors:** Emilia Zicca, Angela Quirino, Nadia Marascio, Stefania Nucara, Fernanda Fabiani, Francesco Trapasso, Nicola Perrotti, Alessio Strazzulla, Carlo Torti, Maria Carla Liberto, Alfredo Focà

**Affiliations:** 1 Institute of Microbiology, Department of Health Sciences, "Magna Graecia" University of Catanzaro, Catanzaro, Italy; 2 Unità Operativa di Genetica Medica, Policlinico Universitario Mater Domini, Università "Magna Græcia" di Catanzaro, Catanzaro, Italy; 3 Dipartimento di Scienze della Salute, Università "Magna Græcia" di Catanzaro, Catanzaro, Italy; 4 Dipartimento di Medicina Sperimentale e Clinica, Università "Magna Græcia" di Catanzaro, Italy; 5 Unit of Infectious Diseases, "Magna Graecia" University of Catanzaro, Catanzaro, Italy; 6 University Unit of Infectious Diseases, University of Brescia, School of Medicine, Brescia, Italy

**Keywords:** HCV, Interleukin 27, SNP, *rs 153109*

## Abstract

**Background:**

Interleukin 27 (IL-27) has pleiotropic properties that can either limit or enhance immune responses. Recent studies revealed that single nucleotide polymorphisms (SNPs) of the IL-27 promoter region modulate the development of infectious diseases and individual's susceptibility to therapeutic response. Little is known about the relationship between IL-27 single nucleotide polymorphisms and therapy response in patients infected by hepatitis C virus (HCV). In this study we have investigated the potential role of SNPs in the promoter region of *IL27 p28 gene *(alleles *rs153109*) on the outcome of HCV infected patients.

**Methods:**

*rs153109*, corresponding to position c.-964A>G of the *IL-27 *locus, was amplified from genomic DNA extracted from 15 patients with chronic hepatitis C stratified by sustained viral response (SVR), relapser and non-responder, after treatment with peginterferon-α (PegIFN- α) combined with ribavirin (RBV). Amplification products were studied by direct sequencing.

**Results:**

This method has been applied in a preliminary study on patients with chronic hepatitis C to provide information for a standardized assay useful to genotyping of *rs153109* SNPs of IL-27p28. The genotype distribution of the c.-964 A>G polymorphism was more present in patients who did not achieve a SVR. By contrast, the genotype G/G was absent in non-responder and relapser patients. Moreover, the analysis of allelic distribution of *rs153109 *highlighted a predominance of allele A in all genotypes in spite of allele G.

**Conclusions:**

Our work provides preliminary information for a standardized method potentially useful for genotyping *rs153109*, and suggests its utility as a candidate approach to evaluate *IL-27 p28 *polymorphisms as additional clinical predictors of response to therapies in HCV infected patients.

## Background

In agreement to the broad role that cytokines play in shaping many aspects of innate and adaptive immunity, there was a concerted effort toward identifying single nucleotide polymorphisms (SNPs) of cytokine associated with human diseases [1].

Interleukin 27 (IL-27) is a novel Interleukin 12 (IL-12) family member, formed by the dimerization of two subunits, Epstein-Barr virus-induced gene 3 (EBI3) and IL-27p28, which engages a receptor composed of gp130 and IL-27Rα activating Janus kinase (JAK)/signal transducer and activator of transcription (STAT) and mitogen activated protein kinase (MAPK) signaling. The human IL-27 gene is located on chromosome 16p11 and consists of five exons. Recently, both c.-964A/G ( *rs153109*) and 2905T/G (*rs 181206*) polymorphisms, corresponding to promoter region of *IL-27p28 *gene, were identified to be associated with individual susceptibility of asthma, inflammatory bowel diseases [2,3]. The findings of Huang *et al*., [4] suggest that polymorphisms of IL-27 gene -964 A/G may not be involved in susceptibility to colorectal cancer (CRC), but this does not exclude the possible involvement of other polymorphisms of IL-27 like 2905 T/G (*rs 181206*). Moreover, Robinson *et al*. [5] showed the ability of IL-27 to regulate the macrophage activity during *Mycobacterium tuberculosis *infection while Peng *et al*., [6] associated SNPs of IL-27 gene with the development of chronic hepatitis B.

Furthermore, recent reports have shown that IL-27 significantly induces interferon (IFN)-inducible antiviral genes, suggesting that IL-27 inhibits human immunodeficiency virus (HIV), influenza virus and hepatitis C virus (HCV) replication by eliciting an IFN-like response [7-10]. The mechanisms underlying the HCV-induced liver damage have not been completely clarified yet. Several studies have shown that hepatitis pathogenesis and the rate of liver disease progression are influenced by several factors such as environmental parameters and viral factors (genotype and level of viremia) [11-15].

However, this approach is considered limitative because the evolution of liver disease and the response to treatment appear to be the result of a dynamic process in which genetic factors of host are mainly involved.

Indeed, information about genetic variants, either mutant or polymorphic, represents the basis for the development of new clinical approach for the management of chronic HCV infected patients [16]. Several independent genome-wide association studies (GWAS) have reported that SNPs near the gene *IL-28B *are strongly correlated with HCV containment, spontaneous clearance, host response to antiviral treatment and disease progression [17-19].

All these findings prompted us to hypothesize that interleukin-27 could influence the HCV infection susceptibility and response to therapy.

In this report, we show the development of an assay useful to detect SNP of the promoter region of *IL27 p28 gene *( *rs153109*) and report preliminary data on HCV RNA-positive patients undergoing treatment with peginterferon-α (PegIFN- α) combined with ribavirin (RBV).

## Methods

### Patients

The study was designed as a retrospective analysis. The sample of individuals studied was composed of 15 patients with chronic hepatitis C. All subjects were treated with Peg-IFN-α-2a at a fixed dose of 180 μg/week or with Peg-IFN-α-2b at 1.5 μgr/kg/week and ribavirin 800-1.200 mg/day (i.e., 800 mg for patients <65 kg; 1.000 mg for patients weighing 65 to 85 kg; 1.200 mg for patients weighing 85 to 105 kg). Patients with HCV genotype 1 were treated for 24-48 weeks and 24 weeks in patients with genotype non-1.

The sample was stratified according to therapeutic response in: sustained viral response (HCV RNA negative 24 weeks after cessation of treatment, SVR), relapser (reappearance of HCVRNA in serum after therapy is discontinued) and non-responder (failure to decrease HCV RNA < 2 log_10 _after 24 weeks of therapy). Baseline characteristics of 15 patients with hepatitis C are summarized in Table 1.

**Table 1:** Baseline characteristics of enrolled patients with HCV infection.

Characteristics	Patients N°(%)
**Gender **	
Male	7 (46.7)
Female	8 (53.3)

**Age **	
Mean	52.7

**HCV genotype **	
1b	12 (80)
Other	3 (20)

**Virogical Response to PEG-INF-α/RBV **	
SVR	5 (33.3)
Relapser	4 (26.7)
Non Responder	6 (40)

**APRI SCORE **	
≤0.5	8 (53.3)
0.51-1.5	2 (13.4)
>1.5	5 (33.3)

**Log_10 _HCV-RNA (UI/ml)**	
Mean	6.16

**Frequencies IL-27 genotypes **	
A/A	5 (33.3)
A/G	9(60)
G/G	1 (6.7)
A Allele	14 (93.3)
**Total**	15

**PegIFN-α**, peginterferon-α; **RBV**, Ribavirin; **SVR**, Sustained Viral Response; **APRI SCORE**, AST to Platelet Ratio Index;

The study was approved by the Ethic Committee of University Hospital Mater Domini, Catanzaro, Italy and the patients signed an informed consent.

### Extraction of genomic DNA, primers design, sequencing analysis of SNP *rs 153109*

Genomic DNA was extracted from peripheral blood leukocyte samples by a Genomic DNA Extraction kit (Nuclear Laser Medicine S.r.l.), according to the manufacturer's directions. DNA quality was assessed by calculating the absorbance ratio OD260nm/280nm using NanoDrop (Thermo scientific 1000 spectrophotometer) and by electrophoresis on 1% agarose gel.

**Figure 1:**
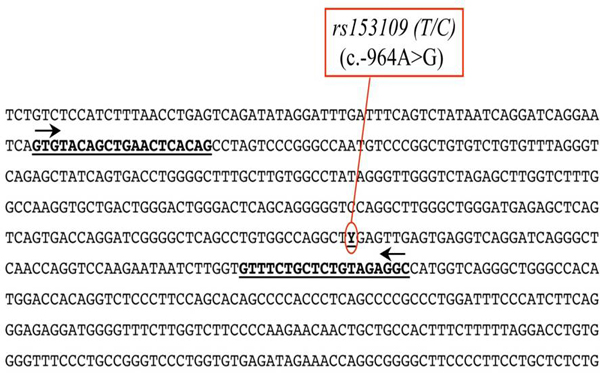
**The nucleotide sequence around *rs153109 *is shown**. Sequences corresponding to forward and reverse primers to determine *rs153109 *polymorphism are underlined.

Primers were designed to amplify *rs 153109; *length of the amplification product was 306bp. The primers used in this study were the following: *rs153109*_F 5'-gTg TACAGCTgAACTCACAg-3' and *rs153109*_R 5'-gCCTCTACAgAgCAgAAAC-3' (see Figure 1).

Polymerase Chain Reaction (PCR) was performed in a thermocycler VERTI 96well (Applied Biosystems) in a total volume of 50 μl containing 150 ng genomic DNA, 25 μM primers, 1,25 U Taq DNA Polymerase, 50 m M KCl, 30 m M Tris- Tris-HCl, 1,5 mM Mg^2+ ^(*MasterMix 2.5 x, 5prime*) and 200 μM dNTP. PCR was carried out under the following thermal cycler conditions: initial denaturation at 95°C for 3 minutes, followed by 38 cycles consisting of denaturation at 95°C for 30 seconds, annealing at 52° C for 30 seconds, extension at 72°C for 40 seconds; a final extension at 72°C for 5 minutes was performed.

**Figure 2:**
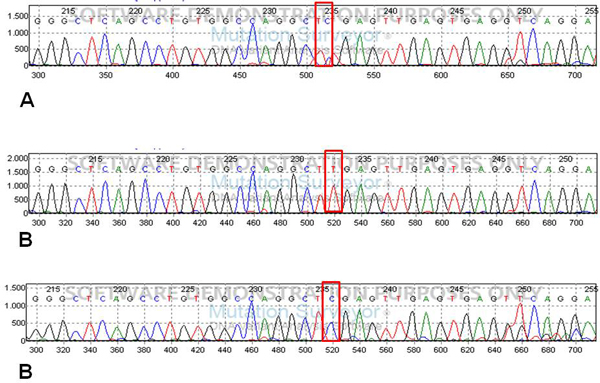
**SNP *rs153109 *direct sequencing**. Representative electropherogram of direct sequencing analysis, heterozygote A/G (**A**) and homozygote A/A e G/G (**B**). The red rectangles indicate the SNP position.

After purification using *PCR Illustra MicroSpin S-300 Colonne HR *(Gelifesciences UK), amplification products were used as template DNA for sequencing analysis (ABI Prism Big Dye Terminator Cycle Sequencing Ready Reaction kit version 1.1 on an ABI PRISM 3100 genetic analyzer [Applied Biosystems]). PCR products were studied by direct sequencing (see Figure 2).

SNP *rs153109 *were detected by sequence analysis based on the reference sequence of *Homo sapiens *chromosome 16 genomic contig GRCh37.p13 Primary Assembly (NCBI Reference Sequence: NT_010393.16).

## Results and future developments

**Figure 3:**
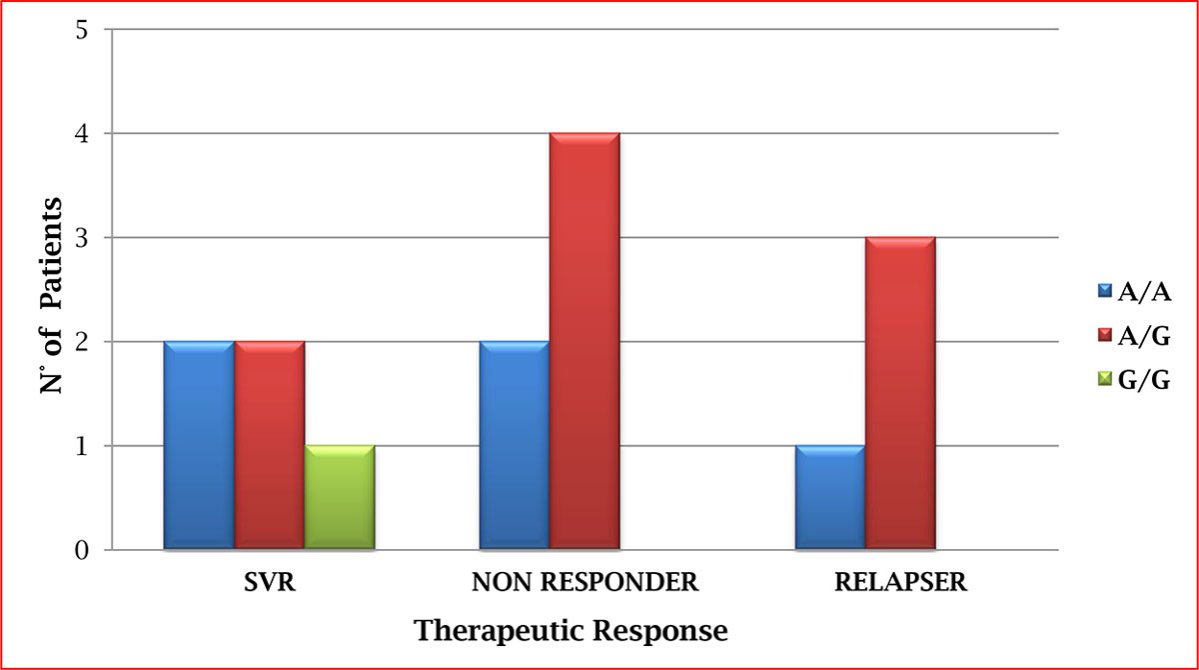
**Distribution of genotype of SNP *rs153109 *of *IL-27p28 *gene**. The eterozygote genotype A/G of SNP is principally present in non-responder and relapser patients compared to subjects with a sustained viral response (SVR). The homozygote genotype G/G was totally absent in patients who respond negatively to antiviral therapy and the analysis of allele frequencies of the SNPs highlights a predominance of allele A in all genotype in spite of allele G.

In this preliminary report, we evaluated the *rs153109 *alleles of the *IL27p28 *gene in patients with chronic hepatitis C using a direct sequencing approach. In particular, we analyzed the genotype of this SNP on c. -964A>G. Our data show a different distribution of genotype and allele of SNP in our patients. Indeed, the A/G genotype is present in non-responders and relapser compared to patients with a sustained viral response (SVR). In addition, the G/G genotype was absent in patients who negatively respond to antiviral therapy. Moreover, the analysis of allele distributions of the SNPs highlights a predominance of allele A in all genotypes in spite of allele G (see Figure 3).

It is well known that host and viral factors can predict treatment outcome; host factor, such as SNPs of Interleukin 28A (IL28A), Interleukin 28B (IL28B), Interleukin 29 (IL 29), interferon-γ (IFN- γ), mannan binding lectin (MBL), Interleukin 10 (IL−10), Interleukin 18 (IL-18), human cytotoxic T-lymphocyte antigen 4 (CTLA 4), Tumor necrosis factor-related apoptosis inducing ligand receptor 1 (TRAIL), Transforming growth factor (TGF-β), Osteopontin, low molecular mass polypeptides (LMP 7), oligoadenylate synthetase (OAS1) genes, insulin resistance, obesity and ethnicity, have been found to modulate treatment response [20]. Moreover, there is still a great effort for discovering new direct-acting inhibitors of HCV that will be used in combination with interferon or without it. For these reasons, further studies on host genetic factors that may predict the treatment outcome of combinational therapies and progression of liver disease are required.

IL-27 is one of the critical cytokines that functions as a mediator between the innate and adaptive immune system. Moreover, IL-27 synergizes with IL-12 to potentiate Interferon-γ (IFN-γ) production by activated naïve T- and Natural Killer (NK) cell populations [21]. Recent studies revealed that SNPs of the IL-27 promoter region modulate the development of infectious diseases and individual's susceptibility to therapeutic response [5,6].

Our work provides information for a standardized method useful to genotyping of *rs153109 *and might suggest an association between treatment response and genetic variability of *IL-27p28 *SNPs.

Given the small number of patients studied, the statistical association demonstrated in this study may have occurred by chance. However, this approach could represent an interesting starting point for future investigations. Therefore, a larger sample size should confirm our results. Moreover, prospective studies should support the causal relationship between the presence of A/G genotype in non-responder and relapser patients, as well as the major presence of A allele. So, could allow us to identify *IL-27p28 *SNPs as novel genetic markers which may correlate with therapy response and, possibly, with liver disease progression in HCV infected patients.

## List of abbreviations

SNP: Single Nucleotide Polymorphism; IL-27: Interleukin 27; IL-12: Interleukin 12; EBI3: Epstein-Barr Virus-Induced Gene 3; JAK: Janus Kinase; STAT: Signal Transducer and Activator of Transcription; MAPK: Mitogen Activated Protein Kinase; IFN: Interferon; HCV: Hepatitis C Virus; GWAS: Genome Wide Association Studies; PegIFN- α-2a: Peginterferon-α-2a; PegIFN- α-2b: Peginterferon-α-2b; RBV: Ribavirin; SVR: Sustained Viral Response; EtBr: Ethidium Bromide; PCR: Polymerase Chain Reaction; IL28A: Interleukin 28A; IL28B: Interleukin 28B; IL29: Interleukin 29; IFN- γ: Interferon-γ; MBL: Mannan Binding Lectin; IL-10: Interleukin 10; IL-18: Interleukin 18; CTLA4: Human Cytotoxic T-Lymphocyte Antigen 4; TRAIL: Tumor Necrosis Factor-Related Apoptosis Inducing Ligand Receptor 1; TGF-β: Transforming Growth Factor; LMP7: Low Molecular Mass Polypeptides; OAS1: Oligoadenylate Synthetase; NK: Natural Killer.

## Competing interests

The authors declare that they have no competing interests.

## Authors' contributions

E.Z., F.T., N.P., C.T., M.C.M., and A.F. designed research; E.Z., N.M., S.N., F.F. performed research; N.M., A.S., and C.T. collected clinical samples; E.Z., A.Q., N.M. F.T., N.P., A.S., C.T., M.C.M., and A.F. analyzed data; E.Z., F.T., N.P., C.T., M.C.M, and A.F wrote the paper.

## Declarations

Publication of this supplement was partly supported by an unrestricted grant provided by Roche. The articles were independently prepared by the authors with no input from Roche. Roche were not involved in selecting the articles for the supplement. The pegylated IFN/pegIFN treatment mentioned in this article is produced by Roche.

This article has been published as part of *BMC Infectious Diseases *Volume 14 Supplement 5, 2014: Proceedings of the Third Workshop of the HCV Study Group in the Calabria Region: Results from the South Italian Network for Rational Guidelines and International Epidemiology (SINERGIE) Project. The full contents of the supplement are available online at http://www.biomedcentral.com/bmcinfectdis/supplements/14/S5.
